# A three-microRNA signature as a diagnostic and prognostic marker in clear cell renal cancer: An *In Silico* analysis

**DOI:** 10.1371/journal.pone.0180660

**Published:** 2017-06-29

**Authors:** Bin Liang, Jianying Zhao, Xuan Wang

**Affiliations:** 1 Department of Bioinformatics, Key Laboratory of Cell Biology, Ministry of Public Health and Key Laboratory of Medical Cell Biology, Ministry of Education, College of Basic Medical Science, China Medical University, Shenyang, China; 2 Department of Clinical Laboratory, No. 202 Hospital of PLA, Shenyang, China; 3 Graduate School, Jinzhou Medical University, Jinzhou, China; 4 Graduate School, Dalian Medical University, Dalian, China; University of South Alabama Mitchell Cancer Institute, UNITED STATES

## Abstract

Accumulating evidence has demonstrated that some specific miRNAs were aberrantly expressed in renal clear cell carcinoma and participated in many biological processes. The aim of this study was to investigate a panel of miRNA signature for diagnosis and prognosis of renal clear cell carcinoma (KIRC). Here, we performed a comprehensive analysis for miRNA expression profiles and corresponding clinical information of 516 KIRC patients from The Cancer Genome Atlas (TCGA). In the study, a total of 63 differentially expressed miRNAs were identified, of which 34 were up-regulated and 29 were down-regulated. We constructed a panel of three-miRNA that were significantly associated with KIRC diagnosis and KIRC patients' prognosis. The three-miRNA signature reached a sensitivity of 98.3% and a specificity of 97.2% in the diagnosis of KIRC. Using the three-miRNA signature, we classified the KIRC patients into high-risk group and low-risk group. The Kaplan- Meier curves showed that KIRC patients with high risk scores had significantly worsen overall survival (OS) and disease free survival (DFS) than KIRC patients with low risk scores. In the univariate and multivariate Cox regression analysis, three-miRNA signature was an independent prognostic factor in OS. In conclusion, the three-miRNA signature could be used as a diagnostic and prognostic biomarker in KIRC, and therefore, may help to provide significant clinical implication for the treatment of KIRC.

## Introduction

Renal cell carcinoma (RCC) is the most lethal urologic cancer, accounting for 2%–3% of adult malignancies in the world [1]. More than 209,000 newly diagnosed RCC and 102,000 deaths caused by RCC are reported per year [2]. Renal clear cell carcinoma (ccRCC) is the frequently observed type of RCC (~80%), which is associated with high morbidity and poor prognosis [3]. While the interactions of environmental factors, genetic and epigenetic alterations on ccRCC development are still unclear, therapeutic options for ccRCCs are still limited [4, 5]. Therefore, understanding how the complex interactions among multiple prognostic factors contribute to the clinical behavior of ccRCC is essential for patient assessment, outcome prediction, and therapy planning.

After the human genome sequencing era, the discovery of an extremely large number of non-coding RNAs conceptually transformed cancer research. MicroRNAs (miRNAs) are small non-protein-coding RNAs consisting of 18–24 nucleotides in length, which regulate gene expression through binding to the 3′ untranslated regions of the mRNAs of target genes, modulating its stability and degradation [6]. A growing body of evidence is emerging to suggest that miRNAs are involved in a wide range of fundamental cellular processes, such as cell differentiation, proliferation, growth, mobility, and apoptosis, as well as carcinogenesis or cancer progression [7]. Several studies have reported that some specific miRNAs were aberrantly expressed in ccRCC and participated in many biological processes. Chen JJ, *et al*. constructed a consistent panel of eleven deregulated miRNAs that can distinguish normal kidney tissues from ccRCC [8]. Heinzelmann J, *et al*. suggested that specific miRNAs are involved in metastasis and have an impact on the progression of the ccRCC [9]. Using high throughput microarray technology, 4-miRNA expression signature was identified to be associated with metastasis, and can determine the metastasis status and predict cancer-related survival in ccRCC patients [10]. Potential mechanisms by which miRNAs contribute to ccRCC pathogenesis are still poorly understood. Therefore, the identification of these related miRNAs may contribute to ccRCC early diagnosis and survival prognosis.

Recently, the Cancer Genome Atlas (TCGA) database (https://cancergenome.nih.gov/) can be used to analyze complicated clinical profiles and cancer genomics. The recent publication of TCGA Kidney Renal Clear Cell Carcinoma (KIRC) project has provided an immense wealth and breadth of data, providing an invaluable tool for confirmation and expansion upon previous observation in a large data set containing multiple data types. In the present study, we screened the differentially expressed miRNAs between KIRC tissues and matched normal tissues, and found the association of different miRNAs expression with clinical characteristics. More importantly, we constructed a three-miRNAs signature that may serve as a potentially diagnostic marker and predictor of prognosis in KIRC.

## Materials and methods

### Data processing

The miRNA sequencing data (level 3) (https://cancergenome.nih.gov/) and corresponding clinical information for 516 KIRC patients were downloaded from the TCGA database (http://www.cbioportal.org/). Table 1 provided the detailed clinical information, including gender, age at diagnosis, tumor size, metastasis status, lymph node status, and TNM stage (according to the seventh edition AJCC). The median follow-up time was 38.96 months (range from 0–149.05 months).

**Table 1:** Clinical characteristics of KIRC patients.

Variables	Case, n (%)
Age at diagnosis	
<60	239 (46.3%)
≥60	277 (53.7%)
Gender	
female	181 (35.1%)
male	335 (64.9%)
Tumor size	
<2cm	348 (72.3%)
≥2cm	133 (27.7%)
Metastasis	
M0	404 (78.6%)
M1	79 (15.4%)
MX	31 (6.0%)
Lymph node status	
N0	228 (44.2%)
N1	16 (3.1%)
NX	272 (52.7%)
Stage	
I	253 (49.3%)
II	55 (10.7%)
III	122 (23.8%)
IV	83 (16.2%)
T stage	
T1	259 (50.2%)
T2	66 (12.8%)
T3	180 (34.9%)
T4	11 (2.1%)

The different expressed miRNAs between KIRC tissues and matched normal tissues were analyzed using the limma package in R. The unpaired *t-*test was used to identify miRNAs that were significantly differentially expressed between KIRC samples and matched normal samples. Fold changes (FCs) in the expression of individual miRNA were calculated and differentially expressed miRNAs with *P*<0.05 and log_2_|FC|>2.0 were considered to be significant.

### Diagnostic performance of differentially expressed miRNAs

The diagnostic performance of differentially expressed miRNAs was evaluated using receiver operating characteristic (ROC) curve. To judge the superiority or inferiority of the miRNAs, area under the ROC curves (AUROC) was determined to establish the diagnostic sensitivity and specificity. *P* < 0.05 was considered statistically significant.

### Association of differentially expressed miRNAs and patient prognosis

All patients were divided into high or low miRNA expression group according to median value. The end point of the present study was overall survival (OS) and disease free survival (DFS). OS was assessed from the day of diagnosis to the day of last follow-up, while DFS was defined as the time from the day of the first complete remission to the day of first relapse or death. The Kaplan-Meier and Log-rank method were used to test the difference in two groups. A *P*-value of less than 0.05 was considered to be significant.

### Target gene prediction of three miRNAs and functional analysis

The target genes of three miRNAs were predicted using two online analysis tools: miRDB (http://www.mirdb.org/miRDB) and TargetScan (http://www.targetscan.org). In order to enhance the bioinformatics analysis reliability, the overlapping target genes were identified using Venn diagram (http://www.venndiagram.net/). The Kyoto Encyclopedia of Genes and Genomes (KEGG) pathways and Gene ontology (GO) were analyzed using The Database for Annotation, Visualization and Integrated Discovery (DAVID) bioinformatics tool (https://david.ncifcrf.gov/).

### Statistical analysis

The data were expressed as mean ± standard deviation (SD). The association between clinicopathological parameters and miRNA expression was evaluated using *x*^2^ tests. The three-miRNA signature was derived from significant miRNAs in OS, DFS and diagnostic performance. The prognostic significance of three-miRNA signature was evaluated by the univariate and multivariate Cox proportional hazard regression model. All statistical analysis was performed by SPSS 22.0 (SPSS Inc., Chicago, IL, USA). All tests were two-sided and *P* <0.05 was considered statistically significant.

## Results

### Identification of differentially expressed miRNAs

**Fig 1:**
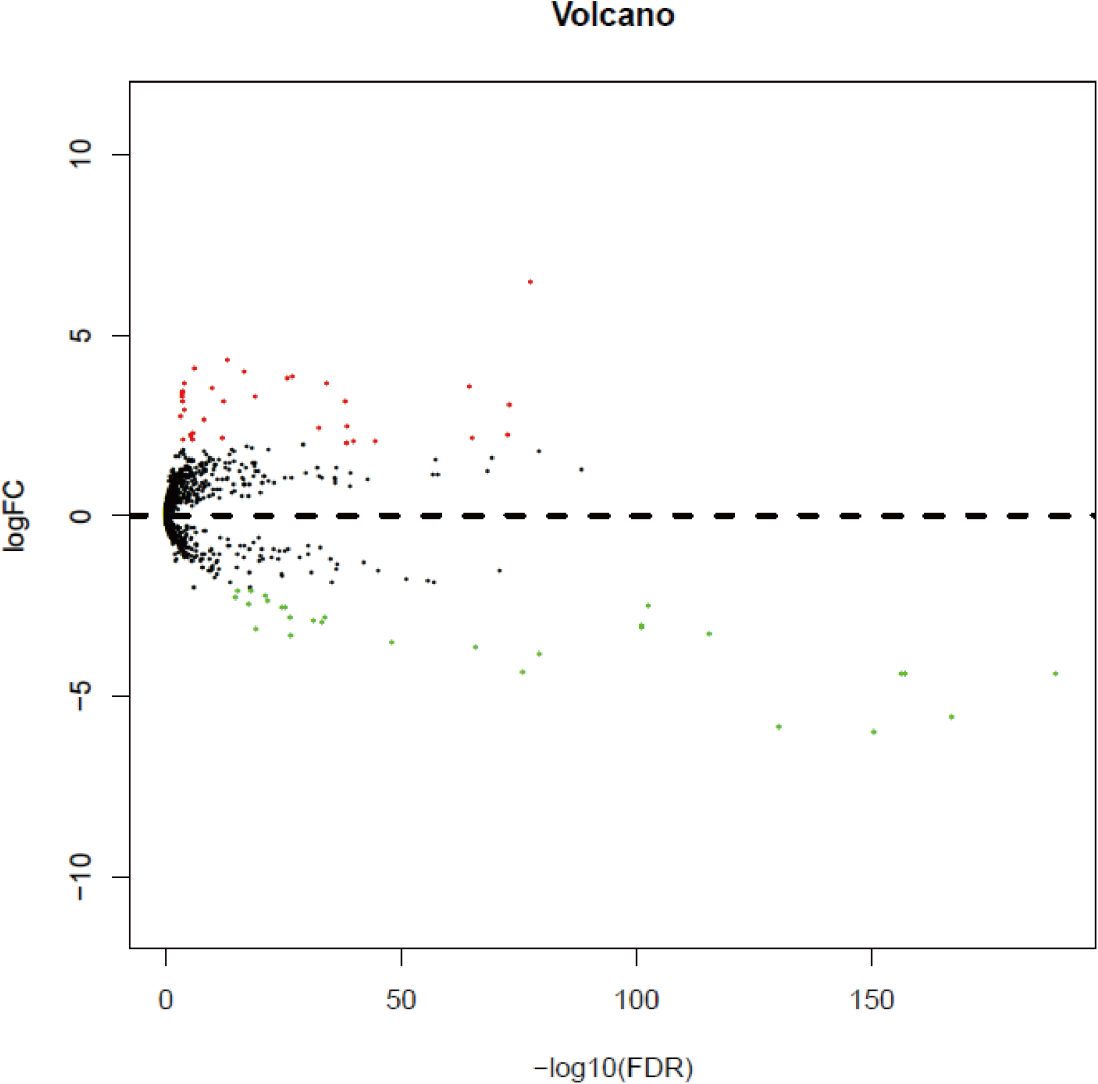
The volcano plot of miRNAs analysis. Plots of log_2_FC vs.–log_10_(FDR) for differentially expressed miRNAs. Red dot represents significant up-regulated miRNA (log_2_|FC|>2.0, *P*<0.05), and green dot represents significant down-regulated miRNA (log_2_|FC|>2.0, *P*<0.05).

With a cut-off value of *P*<0.05 and |log_2_FC| >2.0, a total of 63 differentially expressed miRNAs were identified, of which 34 were up-regulated and 29 were down-regulated (Table 2). In order to prove the *P* value and |log_2_FC| whether conform to logic with different test, the volcano plot was drawn (Fig 1). Unsupervised hierarchic cluster analysis revealed that KIRC tissues could be distinguished from matched normal tissues based on differentially expressed miRNAs patterns (S1 Fig).

**Table 2:** Differentially expressed miRNAs between KIRC tissues and normal tissues.

Up-regulated miRNA				Down-regulated miRNA			
miRNAs	logFC	*P* Value	FDR	miRNAs	logFC	*P* Value	FDR
miR-122	6.46	3.94E-80	4.00E-78	miR-514b	-5.98	1.50E-153	3.80E-151
miR-875	4.30	1.00E-14	1.06E-13	miR-934	-5.83	3.30E-133	7.30E-131
miR-891a	4.08	1.48E-07	9.39E-07	miR-506	-5.59	1.30E-170	9.90E-168
miR-1293	3.99	1.94E-18	2.53E-17	miR-514a-3	-4.40	1.70E-160	8.80E-158
miR-4773-1	3.84	6.55E-29	1.54E-27	miR-514a-1	-4.39	2.40E-160	9.30E-158
miR-4773-2	3.80	6.83E-28	1.49E-26	miR-514a-2	-4.36	1.80E-159	5.40E-157
miR-885	3.66	2.54E-36	7.74E-35	miR-508	-4.36	7.20E-193	1.10E-189
miR-891b	3.66	3.15E-05	0.000145	miR-507	-4.31	1.60E-78	1.53E-76
miR-155	3.58	6.58E-67	4.18E-65	miR-129-1	-3.81	4.47E-82	5.24E-80
miR-599	3.54	2.07E-11	1.81E-10	miR-129-2	-3.63	2.73E-68	1.89E-66
miR-888	3.45	7.09E-05	0.000305	miR-513c	-3.50	2.25E-50	1.11E-48
miR-892a	3.38	0.000107	0.00045	miR-216b	-3.32	1.31E-28	2.98E-27
miR-4652	3.30	9.72E-21	1.50E-19	miR-509-3	-3.25	2.20E-118	4.10E-116
miR-892b	3.29	8.93E-05	0.000379	miR-184	-3.12	5.23E-21	8.30E-20
miR-374c	3.18	5.71E-14	5.84E-13	miR-509-1	-3.07	7.20E-104	1.00E-101
miR-892c	3.18	7.45E-05	0.00032	miR-509-2	-3.06	5.70E-104	8.70E-102
miR-592	3.14	2.23E-40	8.30E-39	miR-200c	-2.93	3.27E-35	9.40E-34
miR-210	3.06	1.14E-75	1.03E-73	miR-513a-1	-2.90	1.61E-33	4.22E-32
miR-1269b	2.94	2.91E-05	0.000135	miR-513a-2	-2.83	2.69E-28	6.02E-27
miR-890	2.76	0.000258	0.001017	miR-510	-2.80	7.20E-36	2.15E-34
miR-4784	2.68	1.21E-09	9.28E-09	miR-372	-2.55	2.35E-27	5.04E-26
miR-224	2.48	8.06E-41	3.15E-39	miR-203b	-2.55	1.38E-26	2.80E-25
miR-3941	2.44	1.34E-34	3.65E-33	miR-362	-2.49	2.30E-105	3.90E-103
miR-3609	2.31	3.47E-07	2.13E-06	miR-513b	-2.43	2.35E-19	3.23E-18
miR-4454	2.24	1.23E-06	6.96E-06	miR-138-1	-2.37	1.30E-23	2.36E-22
miR-21	2.23	2.63E-75	2.23E-73	miR-141	-2.27	2.12E-16	2.49E-15
miR-7641-2	2.19	6.56E-07	3.86E-06	miR-138-2	-2.22	5.04E-23	9.04E-22
miR-3591	2.15	1.26E-13	1.27E-12	miR-6507	-2.07	4.40E-17	5.28E-16
miR-584	2.15	1.59E-67	1.05E-65	miR-1251	-2.07	6.62E-20	9.52E-19
miR-1269a	2.12	5.28E-05	0.000232	miR-514b	-5.98	1.50E-153	3.80E-151
miR-219a-2	2.11	4.10E-07	2.50E-06	miR-934	-5.83	3.30E-133	7.30E-131
miR-4772	2.07	8.36E-47	3.86E-45	miR-506	-5.59	1.30E-170	9.90E-168
miR-142	2.06	3.12E-42	1.32E-40	miR-514a-3	-4.40	1.70E-160	8.80E-158
miR-452	2.03	1.09E-40	4.13E-39	miR-514a-1	-4.39	2.40E-160	9.30E-158
miR-514b	-5.98	1.50E-153	3.80E-151	miR-514a-2	-4.36	1.80E-159	5.40E-157
miR-934	-5.83	3.30E-133	7.30E-131	miR-508	-4.36	7.20E-193	1.10E-189
miR-506	-5.59	1.30E-170	9.90E-168	miR-507	-4.31	1.60E-78	1.53E-76
miR-514a-3	-4.40	1.70E-160	8.80E-158	miR-129-1	-3.81	4.47E-82	5.24E-80
miR-514a-1	-4.39	2.40E-160	9.30E-158	miR-129-2	-3.63	2.73E-68	1.89E-66
miR-514a-2	-4.36	1.80E-159	5.40E-157	miR-513c	-3.50	2.25E-50	1.11E-48
miR-508	-4.36	7.20E-193	1.10E-189	miR-216b	-3.32	1.31E-28	2.98E-27
miR-507	-4.31	1.60E-78	1.53E-76	miR-509-3	-3.25	2.20E-118	4.10E-116
miR-129-1	-3.81	4.47E-82	5.24E-80	miR-184	-3.12	5.23E-21	8.30E-20
miR-129-2	-3.63	2.73E-68	1.89E-66	miR-509-1	-3.07	7.20E-104	1.00E-101
miR-513c	-3.50	2.25E-50	1.11E-48	miR-509-2	-3.06	5.70E-104	8.70E-102
miR-216b	-3.32	1.31E-28	2.98E-27	miR-200c	-2.93	3.27E-35	9.40E-34
miR-509-3	-3.25	2.20E-118	4.10E-116	miR-513a-1	-2.90	1.61E-33	4.22E-32
miR-184	-3.12	5.23E-21	8.30E-20	miR-513a-2	-2.83	2.69E-28	6.02E-27
miR-509-1	-3.07	7.20E-104	1.00E-101	miR-510	-2.80	7.20E-36	2.15E-34
miR-509-2	-3.06	5.70E-104	8.70E-102	miR-372	-2.55	2.35E-27	5.04E-26
miR-200c	-2.93	3.27E-35	9.40E-34	miR-203b	-2.55	1.38E-26	2.80E-25
miR-513a-1	-2.90	1.61E-33	4.22E-32	miR-362	-2.49	2.30E-105	3.90E-103
miR-513a-2	-2.83	2.69E-28	6.02E-27	miR-513b	-2.43	2.35E-19	3.23E-18
miR-510	-2.80	7.20E-36	2.15E-34	miR-138-1	-2.37	1.30E-23	2.36E-22
miR-372	-2.55	2.35E-27	5.04E-26	miR-141	-2.27	2.12E-16	2.49E-15
miR-203b	-2.55	1.38E-26	2.80E-25	miR-138-2	-2.22	5.04E-23	9.04E-22
miR-362	-2.49	2.30E-105	3.90E-103	miR-6507	-2.07	4.40E-17	5.28E-16
miR-513b	-2.43	2.35E-19	3.23E-18	miR-1251	-2.07	6.62E-20	9.52E-19
miR-138-1	-2.37	1.30E-23	2.36E-22				
miR-141	-2.27	2.12E-16	2.49E-15				
miR-138-2	-2.22	5.04E-23	9.04E-22				
miR-6507	-2.07	4.40E-17	5.28E-16				
miR-1251	-2.07	6.62E-20	9.52E-19				

### Association between differentially expressed miRNAs and clinical characteristics

The differentially expressed miRNAs were further analyzed according to the expression levels and clinical characteristics. The results suggested that miR-210, miR-4772, miR-592, miR-1269a, and miR-203b were linked to age, miR-122, miR-21, miR-584, miR-155, miR-142, miR-224, miR-875, miR-599, miR-892b, miR-514a-2, and miR-1251 were linked to gender, miR-142, miR-4784, miR-1269b were linked to tumor size, miR-21, miR-584, miR-155, miR-142, miR-885, miR-1293, miR-1269a, miR-1269b, miR-509-2, and miR-1251 were linked to metastasis status, miR-122, miR-210, miR-21, miR-592, miR-885, miR-374c, miR-3591, miR-200c, miR-1251, and miR-141 were linked to lymph node status. Moreover, we also found that 17 miRNAs were associated with clinical stage and 19 miRNAs were associated with T stage (Table 3).

**Table 3:** Association between differentially expressed miRNAs and clinical characteristics.

Variables	Up-regulated miRNAs	Down-regulated miRNAs
Age (<60 vs.≥60)	miR-210, miR-4772, miR-592, miR-1269a	miR-203b
Gender (female vs. male)	miR-122, miR-21, miR-584, miR-155, miR-142, miR-224, miR-875, miR-599, miR-892b	miR-514a-2, miR-1251
Tumor size (<2cm vs.≥2cm)	miR-142, miR-4784, miR-1269b	
Metastasis (M0 vs. M1)	miR-21, miR-584, miR-155, miR-142, miR-885, miR-1293, miR-1269a, miR-1269b	miR-509-2, miR-1251
Lymph node status (N0 vs. N1)	miR-122, miR-210, miR-21, miR-592, miR-885, miR-374c, miR-3591	miR-200c, miR-1251, miR-141
Stage (I+II vs. III+IV)	miR-21, miR-584, miR-155, miR-142, miR-224, miR-885, miR-1293, miR-875, miR-219a-2, miR-1269a, miR-1269b	miR-514a-2, miR-362, miR-509-2, miR-129-1, miR-203b, miR-1251
T stage (T1+T2 vs.T3+T4)	miR-21, miR-584, miR-155, miR-142, miR-224, miR-885, miR-4773-1, miR-4773-2, miR-1293, miR-219a-2, miR-1269a, miR-1269b	miR-514a-2, miR-362, miR-509-2, miR-129-1, miR-203b, miR-138-2, miR-1251

### Diagnostic performance of differentially expressed miRNAs

**Fig 2:**
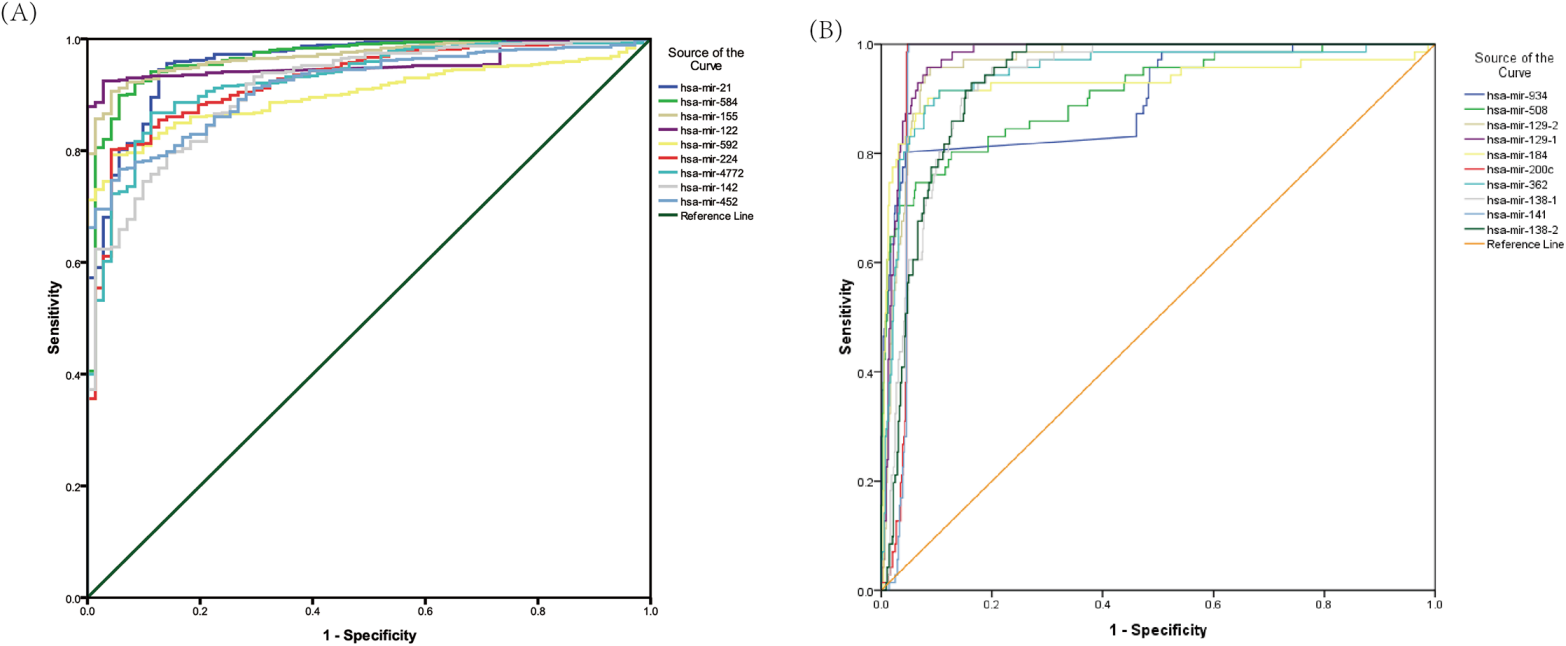
ROC curves of miRNAs in discriminating KIRC from normal controls. (A) up- regulated miRNAs (AUROC>0.9); (B) down-regulated miRNAs (AUROC>0.9).

In order to evaluate the discriminatory values of differentially expressed miRNAs between KIRC and matched normal tissues, we performed ROC analysis. The AUROC ranges from 0.90–1.0, which is considered to be "excellent" at separating disease status from controls. The up-regulated miRNAs, miR-21, miR-584, miR-155, miR-122, miR-592, miR-224, miR-142, miR-452, and miR-4772 had good diagnostic performances, with the AUROC of 0.958, 0.966, 0.969, 0.956, 0.901, 0.924, 0.912, 0.916, and 0.925 (Fig 2A). In addition, the down-regulated miRNAs, miR-934, miR-508, miR-129-1, mir-129-2, miR-184, miR-200c, miR-362, miR-138-1, miR-138-2, and miR-1414 displayed high diagnostic performances, with the AUROC of 0.900, 0.907, 0.973, 0.963, 0.930, 0.959, 0.948, 0.929, 0.931, and 0.957 (Fig 2B). The sensitivity and specificity were showed in Table 4.

**Table 4:** The diagnostic performance of specific miRNAs (AUROC>0.9).

	Variable(s)	AUROC	*P* value	Sensitivity (%)	Specificity (%)
Up-regulated	hsa-mir-21	0.958	<0.001	94.5	87.3
	hsa-mir-584	0.966	<0.001	94.1	88.7
	hsa-mir-155	0.969	<0.001	92.3	93.0
	hsa-mir-122	0.956	<0.001	92.5	97.2
	hsa-mir-592	0.901	<0.001	74.5	97.2
	hsa-mir-224	0.924	<0.001	80.2	95.8
	hsa-mir-142	0.912	<0.001	74.5	90.1
	hsa-mir-452	0.916	<0.001	74.7	95.8
	hsa-mir-4772	0.925	<0.001	83.1	90.1
Down-regulated	hsa-mir-934	0.900	<0.001	80.3	95.6
	hsa-mir-508	0.907	<0.001	76.1	90.8
	hsa-mir-129-2	0.963	<0.001	94.4	92.1
	hsa-mir-129-1	0.973	<0.001	95.8	91.7
	hsa-mir-184	0.930	<0.001	91.5	89.5
	hsa-mir-200c	0.959	<0.001	98.6	95.4
	hsa-mir-362	0.948	<0.001	91.5	89.5
	hsa-mir-138-1	0.929	<0.001	91.5	84.4
	hsa-mir-141	0.957	<0.001	98.6	95.2
	hsa-mir-138-2	0.931	<0.001	93.0	83.7

### Prognostic performance of differentially expressed miRNAs

**Fig 3:**
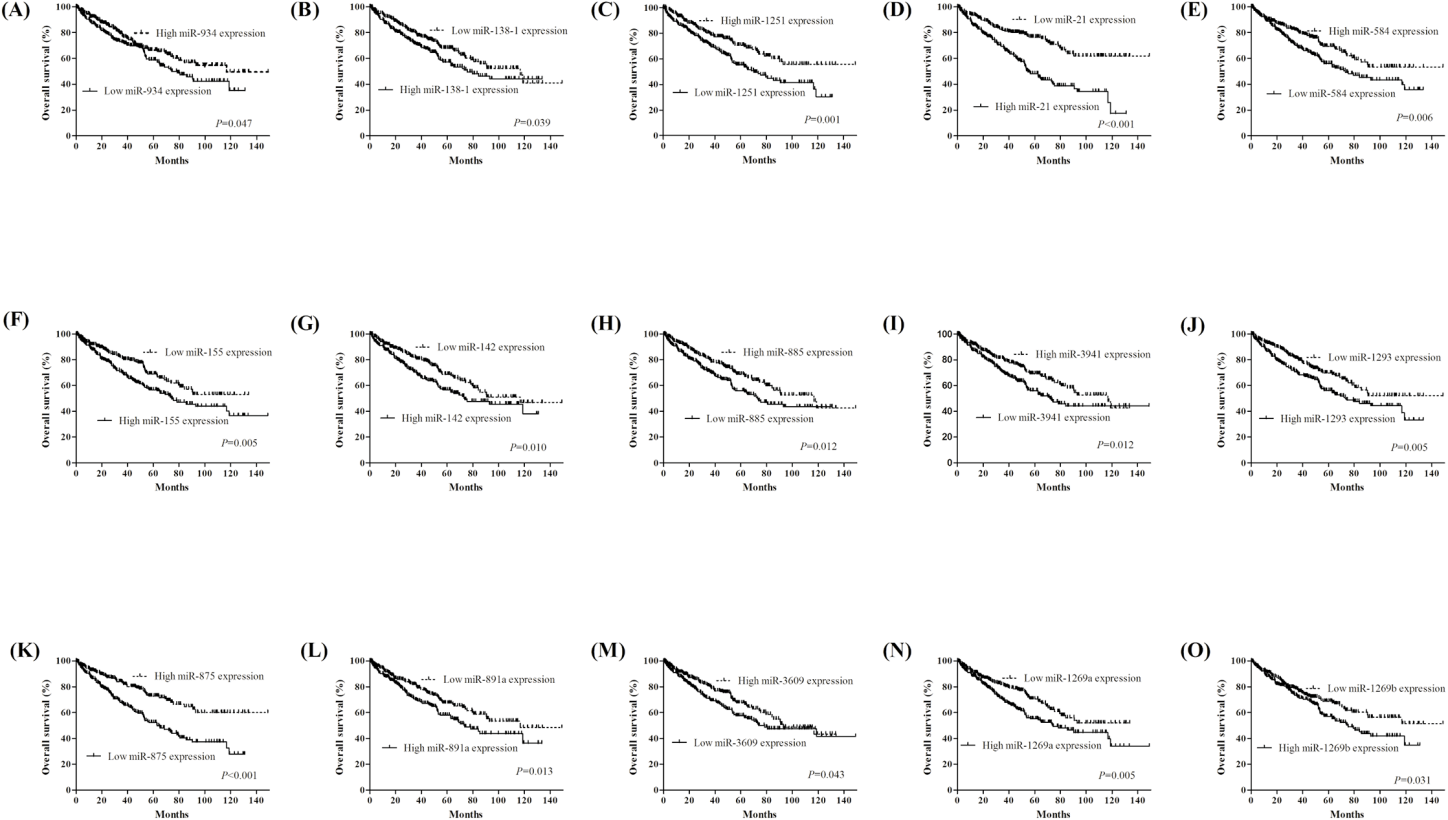
Kaplan-Meier survival curves for 15 miRNAs associated with overall survival.

**Fig 4:**
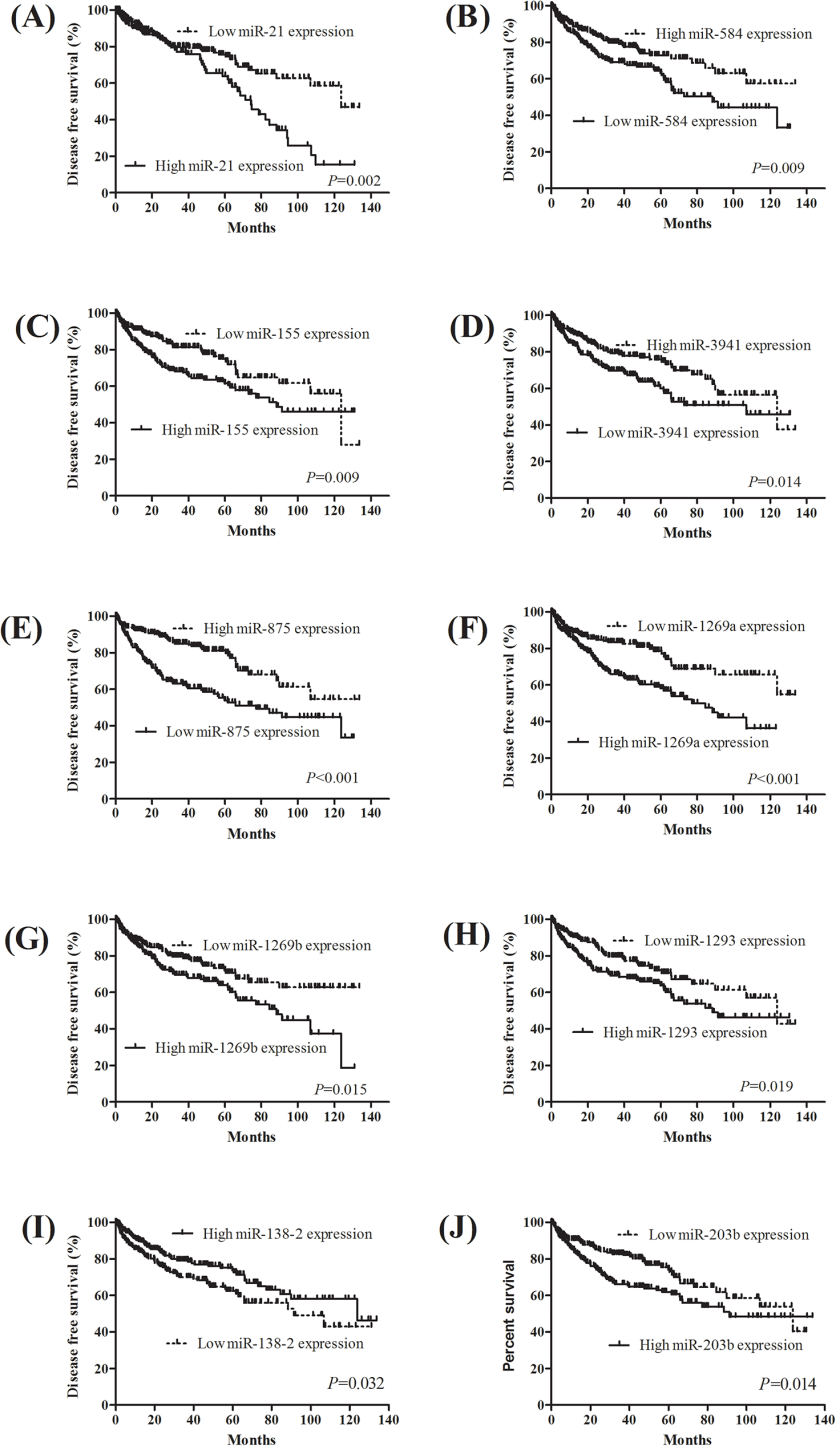
Kaplan-Meier survival curves for 10 miRNAs associated with disease free survival identification of three-miRNA signature in KIRC diagnosis and prognosis.

To explore the prognostic value of miRNAs expression in KIRC, we evaluated the association between miRNAs expression and patients’ survival using Kaplan-Meier analysis with the Log-rank test. In 516 KIRC patients, we found that 15 miRNAs were significantly associated with KIRC patients’ OS (all P<0.05). The 15 miRNAs: miR-21 (*P*<0.001), miR-584 (*P* = 0.006), miR-155 (*P* = 0.001), miR-142 (*P* = 0.010), miR-885 (*P* = 0.012), miR-3941 (*P* = 0.012), miR-1293 (*P* = 0.005), miR-875 (*P*<0.001), miR-891a (*P* = 0.013), miR-3609 (*P*<0.043), miR-1269a (*P* = 0.005), miR1269b (*P* = 0.031), miR-934 (*P* = 0.047), miR-138 (*P* = 0.039), and miR-1251 (*P* = 0.001) were significantly correlated with OS (Fig 3). Next, we evaluate the impact of miRNAs expression on DFS in KIRC patients. The survival plots showed that miR-21 (*P* = 0.002), miR-584 (*P* = 0.009), miR-155 (*P* = 0.009), miR-3941 (*P* = 0.014), miR-875 (*P*<0.001), miR-1269a (*P*<0.001), miR-1269b (*P* = 0.015), miR-1293 (*P* = 0.019), miR-138-2 (*P* = 0.032), and miR-203b (*P* = 0.014) expression had significantly correlated with DFS (Fig 4).

**Fig 5:**
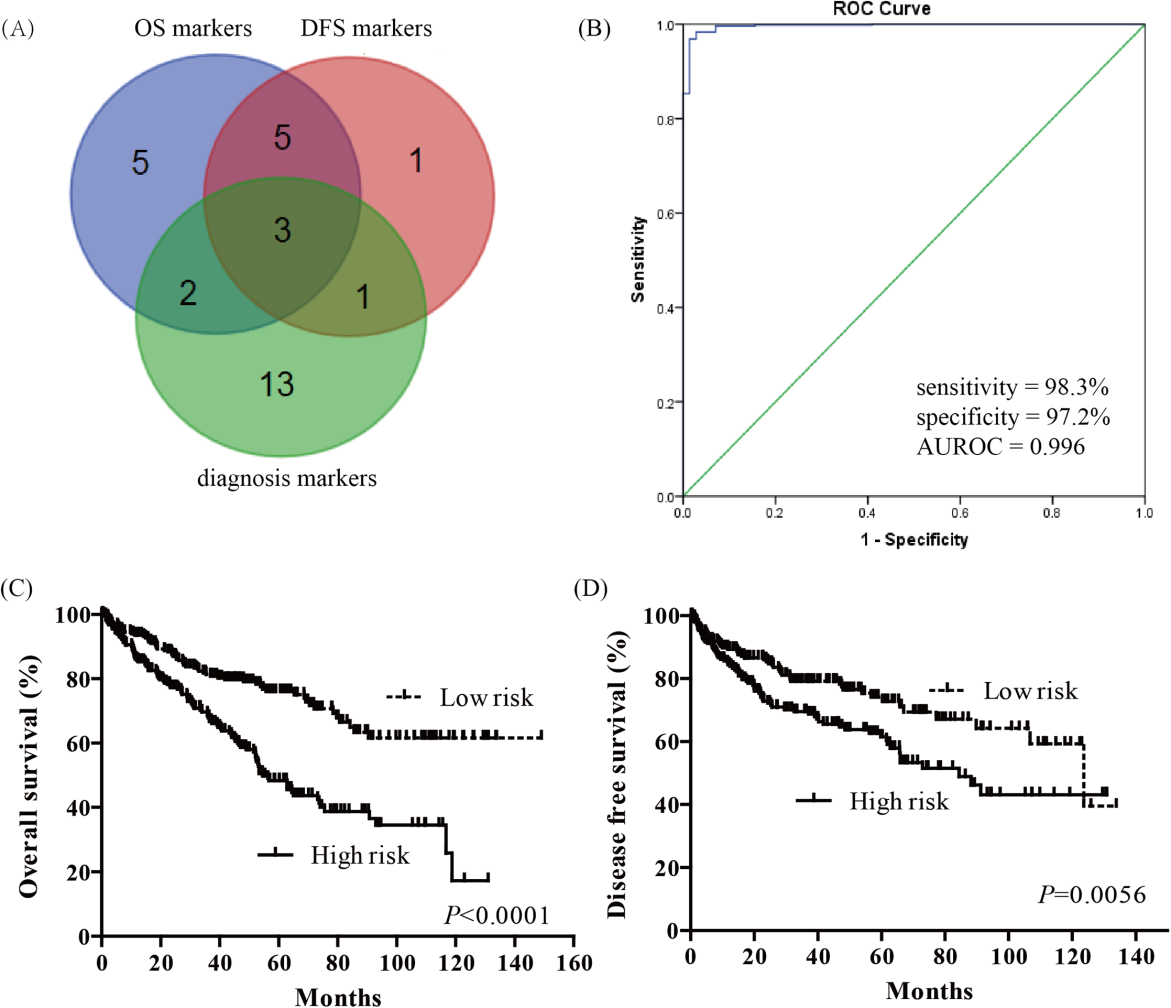
Three-miRNA signature in diagnosis and prognosis of KIRC. (A) Venn analysis of overlapping significant miRNAs among overall survival markers, disease free survival markers and diagnostic markers; (B) ROC curves of three-miRNA signature in differentiating KIRC tissues from normal tissues; (C) Kaplan-Meier survival curves of three-miRNA signature in overall survival prediction; (D) Kaplan-Meier survival curves of three-miRNA signature in disease free survival prediction.

In order to screen diagnostic and prognostic sensitive miRNAs in KIRC patients, we used Venn diagram to identify the overlapping miRNAs. The three-miRNA signature: miR-21, miR-584, and miR-155, was derived from significant miRNAs in OS, DFS and diagnostic performance (Fig 5A). The diagnostic performance of three-miRNA signature was evaluated by ROC curve. The AUROC of three-miRNA signature was 0.996 (95% CI = 0.992–1.000), with a sensitivity of 98.3% and a specificity of 97.2% (Fig 5B). Then, we analyzed the three-miRNA prognosis using a multivariate Cox regression analysis, calculated a risk score for each patient, and ranked them according to increased scores. Thus, the KIRC patients were classified into a high risk group and a low risk group according to the median risk score. The Kaplan-Meier curve showed that patient with high risk scores had significantly worsen OS (*P*<0.0001) and DFS (*P* = 0.0056) than KIRC patients with low risk scores (Fig 5C and 5D). In the univariate and multivariate Cox regression analysis, three-miRNA signature was an independent prognostic factor in OS (HR = 1.980, 95%CI = 1.277–3.077, *P* = 0.002, Table 5).

**Table 5:** Univariate and multivariate Cox regression analysis in KIRC patients.

	Univariate analysis		Multivariate analysis	
	HR (95% CI)	*P* value	HR (95% CI)	*P* value
**Overall survival**				
Gender (male vs. female)	1.078 (0.791–1.470)	0.634		
Age (≥60 vs. <60)	1.859 (1.355–2.550)	<0.001	1.558 (1.010–2.404)	0.045
Tumor size (≥2cm vs. <2cm)	1.470 (1.078–2.005)	0.015		
Mestasis (M1 vs. M0)	4.236 (3.102–5.784)	<0.001	2.915 (1.828–4.651)	<0.001
Lymph node status (N1-2 vs. N0)	3.283 (1.784–6.041)	<0.001	1.898 (1.199–2.994)	0.001
Clinical stage (III+IV vs. I+II)	3.829 (2.782–5.270)	<0.001		
T stage (T3+T4 vs. T1+T2)	3.079 (2.270–4.176)	<0.001		
Three-miRNA signature (high risk vs. low risk)	2.331(1.706–3.185)	<0.001	1.980 (1.277–3.077)	0.002
**Disease free survival**				
Gender (male vs. female)	0.723 (0.487–1.075)	0.109		
Age (≥60 vs. <60)	0.673 (0.471–0.961)	0.029		
Tumor size (≥2cm vs. <2cm)	0.651 (0.447–0.948)	0.025		
Mestasis (M1 vs. M0)	8.264 (5.650–12.048)	<0.001	3.247 (1.883–5.618)	<0.001
Lymph node status (N1-2 vs. N0)	3.476 (1.646–7.339)	0.001	2.532 (1.189–5.405)	0.016
Clinical stage (III+IV vs. I+II)	3.476 (1.646–7.339)	0.001		
Clinical stage (T3+T4 vs. T1+T2)	6.192 (4.166–9.203)	<0.001	3.937 (2.088–7.407)	<0.001
T stage (T3+T4 vs. T1+T2)	4.379 (3.031–6.326)	<0.001		
Three-miRNA signature (high risk vs. low risk)	1.653 (1.154–2.367)	0.006		

### Target genes prediction and functional enrichment analysis

**Fig 6:**
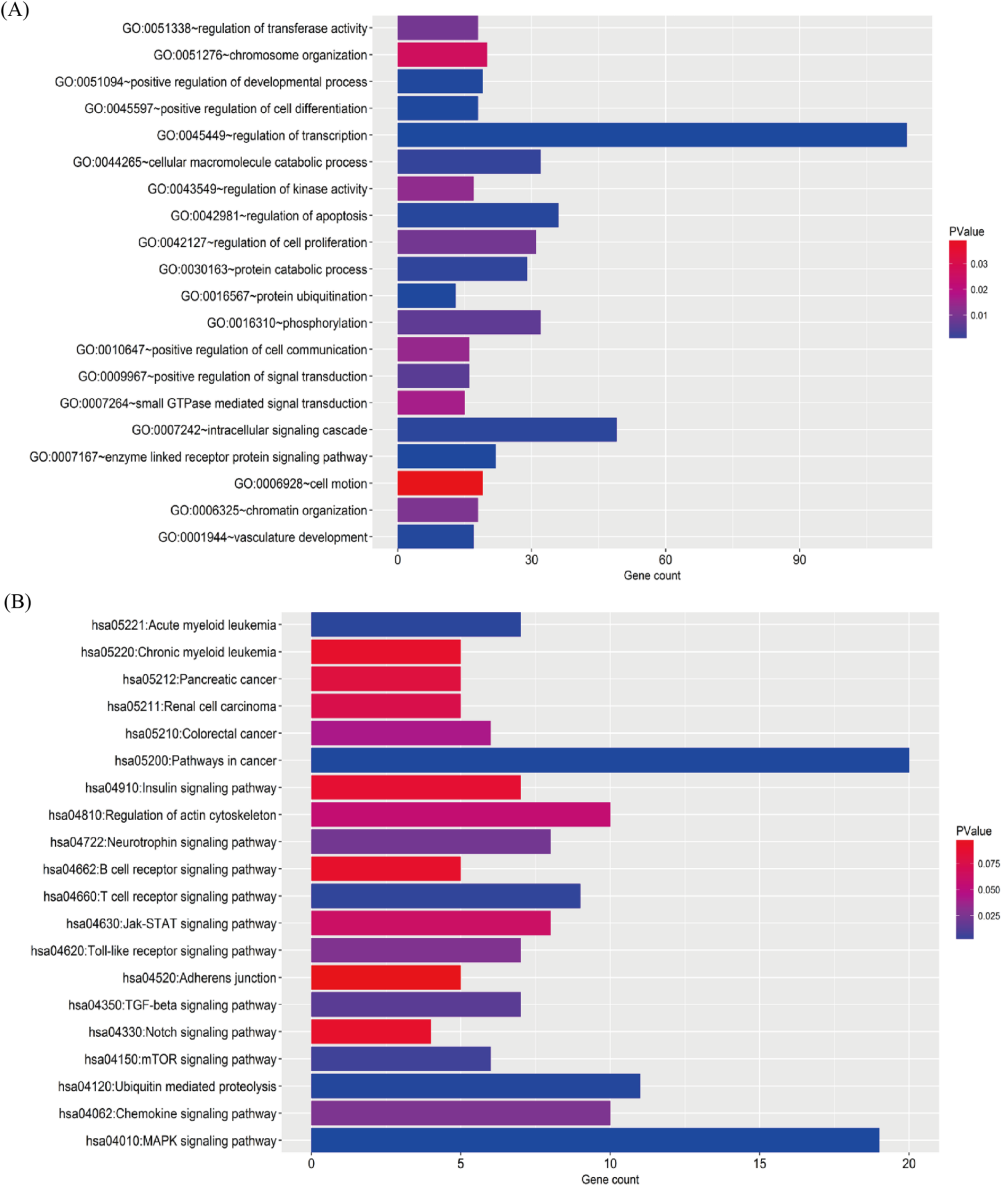
Biological function and KEGG pathway analysis of target genes. The overlapping target genes were predicted using TargetScan and miRDB online analysis tools. (A) the enriched GO biological processes of target genes (B). the enriched KEGG pathways of target genes.

The overlapping target genes were list in supplementary S1 Table. To elucidate the biological processes (BP) and KEGG pathways, we performed enrichment analysis using overlapping target genes. The GO BP involved many processes, including transcription, signaling cascade, apoptosis, macromolecule catabolic process, cell proliferation, phosphorylation, and so on (Fig 6A). The enrichment KEGG pathways were mainly associated with MAPK signaling pathway, ubiquitin mediated proteolysis, T cell receptor signaling pathway, TGF-beta signaling pathway, chemokine signaling pathway, regulation of actin cytoskeleton, and Jak-STAT signaling pathway (Fig 6B).

## Discussion

MiRNAs are considered to be a novel group of disease biomarkers due to the stability and universality in human tissues [11, 12]. Recently, many studies have reported specific miRNA profiles in KIRC, highlighting the roles of miRNAs in the progression of KIRC [13–20]. In the present study, we comprehensively analyzed the miRNA sequencing data downloaded from TCGA datasets. Finally, we identified 63 differentially expressed miRNAs, of which 34 were up-regulated and 29 were down-regulated. We evaluated the diagnostic and prognostic values of each differentially expressed miRNA. A previous study suggested that a multiple miRNA-based signature can provide a more statistically robust analysis than individual miRNA. Accordingly, we developed a three-miRNA signature with excellent diagnostic performance and independent prognostic significance for KIRC patients.

Previous studies have reported that some specific miRNAs were aberrantly expressed in RCC and participated in the development of RCC [21–26]. However, due to clinical and molecular heterogeneity in different studies, as well as methodological difference regarding to reproducibility and normalization, there exists a limitation to identify the specific miRNAs as potential diagnostic and prognostic markers [27]. In addition, the number of patients enrolled in each study is generally small. TCGA, the resource of “big data”, makes gene expression data in tumors and normal tissues available. Translating the data information into a better understanding of underlying biological mechanisms is of importance to identify diagnostic and prognostic markers for KIRC [28].

The recent study retrieved TCGA data and reported that nine high miRNAs expressions were related worse outcome, and 13 high miRNAs expressions were related to better outcome using univariate Cox regression analysis [29]. In addition, Yann Christinat’ study unveiled a novel ccRCC-specific 5-miRNA (miR-10b, miR-21, miR-143, miR-183, and miR-192) signature able to prognosticate ccRCC outcome more accurately than TNM staging alone using a computational approach [30]. In the present study, we analyzed TCGA data using the limma package in R and finally identified 63 differentially expressed miRNAs with *P*<0.05 and |log_2_FC| >2.0. Literature mining confirmed that some of these miRNAs have been reported to be deregulated in RCC, which lends credibility to our list. Gowrishankar B, *et al*. reported that miR-210, miR-21 and miR-142 were up-regulated, and miR-141 and miR-200c were down-regulated in ccRCC compared with normal renal parenchyma [31]. He H, *et al*. suggested that miR-452, miR-200c, miR-155, and miR-142 were commonly dysregulated between ccRCC and adjacent normal tissues [32]. Chen J, *et al*. retrieved a set of 11 miRNAs, which were overlapped by six miRNAs in our study [8]. Shu X, *et al*. reported that miR-155 and miR210 were up-regulated, and miR-141 and miR-200c were down-regulated in tumor-normal comparison [13]. Our findings support a role for these miRNAs in the development of ccRCC.

To explore a potential biomarker in diagnosis and prognosis, we used Venn diagram to indentify a three-miRNA signature: miR-21, miR-584, and miR-155, which were derived from significant miRNAs in OS, DFS and diagnostic performance. Each of the three miRNAs had been previously reported to be associated with many types of cancers, as well as patient survival. miR-21, located on chromosome 17q23.2, is an abundantly expressed miRNA in mammalian cells, and has been shown to be the most commonly upregulated miRNA in solid and hematological malignancies [33]. Emerging evidence has demonstrated that miRNA-21 act as an oncogene by targeting many tumor suppressor genes related to proliferation, apoptosis, and invasion [21]. Recently, Liang T, *et al*. reported that miRNA-21 promoted proliferation and differentiation and decreased apoptosis of human RCC cells through the activation of the mTOR-STAT3 signaling pathway [19]. In addition, miR-21 was also reported to be associated with clinical stage and served as an unfavorable predictor in prognosis of renal cancer [34]. As for miR-155, previous studies indicate that it functions as an oncogenic miRNA in several types of cancer, including breast [35], colon [36], bladder [37], liver [38], and kidney [39]. Shinmei S, *et al*. suggested that miRNA-155 was overexpressed in ccRCC tissues compared with normal kidney tissues [40]. In addition, controversial roles of miR-584 were found in the development and progress of ccRCC. Ueno K, *et al*. reported that miR-584 functions as a tumour suppressor, directly targets oncogene *ROCK-1*, and decreases cell motility in RCC cell lines. But, in our study, we found miR-584 was up-regulated in KIRC tissues, and low miR-584 expression was associated with worse prognosis. Thus, the conflicting function of miR-584 is needed to be further investigated [41]. Here, we performed ROC curve to verify that three-miRNA signature was a potential diagnostic marker in discriminating KIRC from normal controls, reaching a sensitivity to 98.3% and a specificity to 97.2%. Moreover, three-miRNA signature predicted survival better in KIRC, indicating the three-miRNA signature may be a potential predictor of prognosis in KIRC.

However, there are some limitations in our study. First, we are lack of cross-validation of different KIRC patient cohort. Future studies using independent cohorts of large samples from different sample types and multiple institutions are needed to validate our findings for clinical practice. Second, considering that the microarray based studies have identified large numbers of deregulated miRNAs in different renal disease, including diabetic nephropathy, renal fibrosis, polycystic kidney disease, and lupus nephritis [42], further studies should screen the differentially expressed miRNAs between KIRC and other renal diseases. In addition, functional studies of candidate miRNAs in the progression of KIRC should be performed.

## Conclusion

Taken together, by performing a comprehensive analysis for differentially expressed miRNA profiles and corresponding clinical information, our study suggested that three-miRNA signature was a potential diagnostic marker in KIRC, and was an independent prognostic factor in KIRC patients. However, further studies are needed to verify our findings and establish the molecular mechanism for the interplay of miRNAs, their target genes, and KIRC progression.

## Supporting information

S1 Fig Hierarchical clustering of KIRC tissue and matched normal tissues by differentially expressed miRNAs. Each row represents the expression level of a miRNA, and each column represents a sample.
(TIF)

S1 Table Overlapping target genes of three miRNAs using TargetScan and miRDB online tools. (DOCX)
