# Supplementary figures and images for: A three-microRNA signature as a diagnostic and prognostic marker in clear cell renal cancer: An In Silico analysis
Source: PLoS One. 2017 Jun 29;12(6):e0180660. doi: 10.1371/journal.pone.0180660 (PMC5491330; doi:10.1371/journal.pone.0180660)

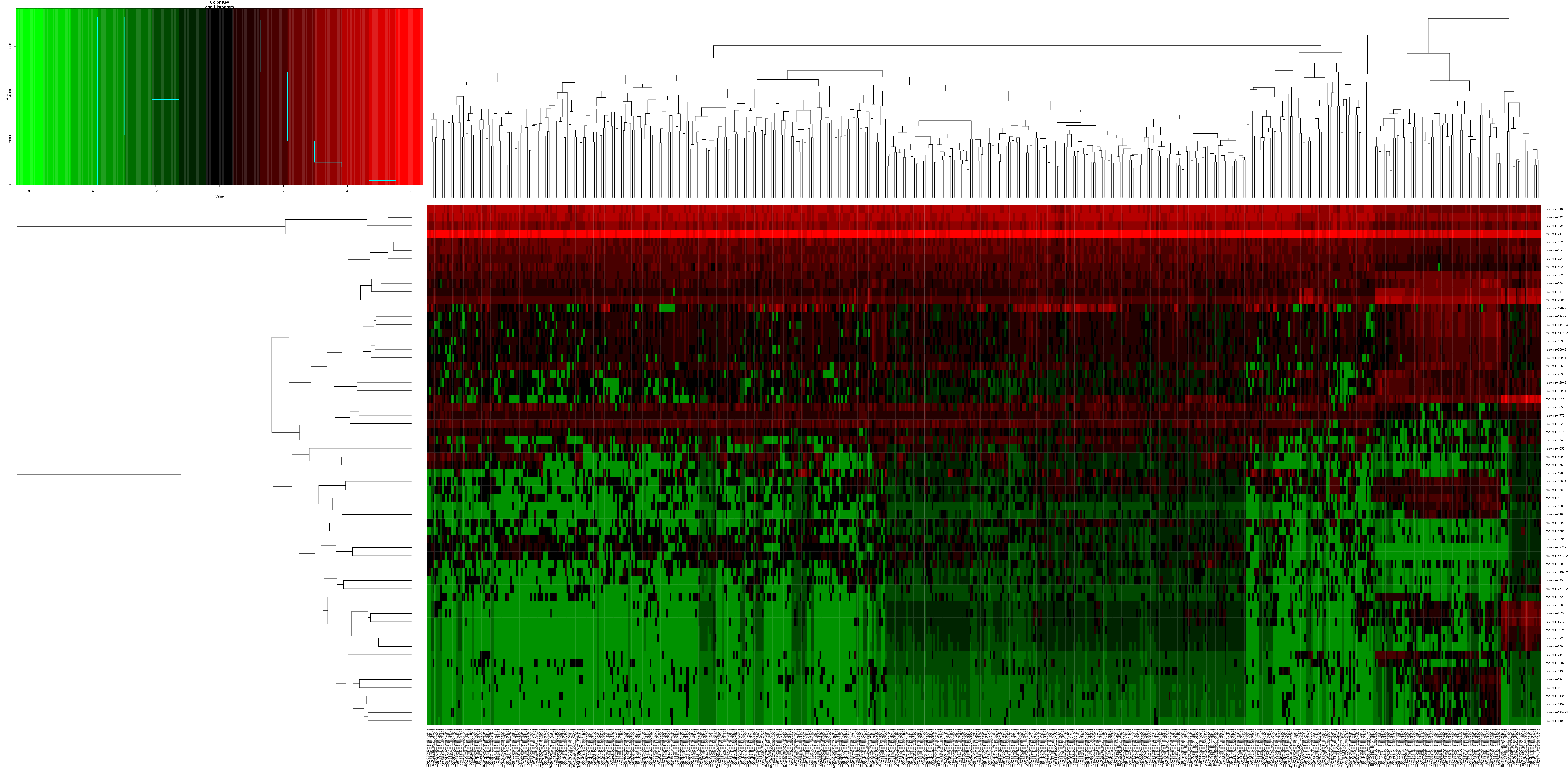

Supplement: S1 Fig — Each row represents the expression level of a miRNA, and each column represents a sample. (TIF) [file pone.0180660.s001.tif]
