# Supplementary material for: A three-microRNA signature as a diagnostic and prognostic marker in clear cell renal cancer: An In Silico analysis
Source: PLoS One. 2017 Jun 29;12(6):e0180660. doi: 10.1371/journal.pone.0180660 (PMC5491330; doi:10.1371/journal.pone.0180660)
Supplement: S1 Table — (DOCX) [file pone.0180660.s002.docx]

**Table S1. Overlapping target genes of three miRNAs using TargetScan and miRDB online tools**

| miRNA | Numbers | Target genes |
| --- | --- | --- |
| miR-21 | 121 | TAGAP SLMAP VCL FASLG XKR6 PITX2 MATN2 CHIC1 PCBP1 UNC80 RMND5A EHD1 AGO2 MTMR12 SCRN1 SMAD7 KRIT1 PIKFYVE SASH1 PPP1R3B NFIA C10orf12 BMP3 BMPR2 CBX4 PIK3R1 TIMP3 STAT3 STAG2 CCL20 ZNF704 ARHGEF12 GTPBP1 RASGRP1 YOD1 PDCD4 SPRY1 DMRTC1B YAP1 UBE2D3 NFIB BCL7A PCBP2 ALX1 RBPJ TRAPPC8 MBNL1 FBXO11 PLEKHA1 FAM46A TESK2 DCAF7 OSR1 PLAG1 UBR3 ARHGAP24 JPH1 ZSWIM6 LRRC57 PTPN14 CPEB3 SKP2 SKI HIPK3 KBTBD6 PFKM PAG1 THRB SCML2 SLC30A10 GATAD2B FBXO28 RECK PELI1 ARMCX1 FGF18 FGD4 DAG1 TGFBI ZNF367 ALX4 MAP2K3 MAP3K1 RALGPS2 NTF3 IL6R JAG1 IL12A SUZ12 PPP1R3A EPHA4 CCL1 ELF2 CNOT6 WWP1 STK40 SPRY2 GPR64 SATB1 GRAMD3 PCSK6 CASKIN1 PAN3 LANCL1 MSH2 TIAM1 FAM13A ZFP36L2 AP1AR PBRM1 PHF14 MBNL3 DUSP8 MEF2C SOX6 KLF6 GLCCI1 PAIP2B KLF3 MBLAC2 BEST3 |
| miR-155 | 154 | CARHSP1 MYBL1 WBP1L LCORL VEZF1 KDM5B TOMM20 H3F3A G2E3 SMAD2 SLC35F5 TRIM23 IRF2BP2 DNAJB1 PTPRD RORA STXBP5L ATAD2B C5orf64 FAM105A HIVEP2 G3BP2 SLC2A12 FBXO22 SCG2 SPI1 GNAS BCORL1 RREB1 CBL ZBTB38 RAPH1 CNTN4 S1PR1 CLCN5 SMUG1 NKX3-1 ARL5B FBXO11 YWHAE ANKFY1 LSM14A FOS C8orf44-SGK3 MIER3 SMARCA4 TP53INP1 TSHZ3 TCEB1 TENM3 AICDA TMEM202 ACTL7A JARID2 RAB11FIP2 KDM2A FAM135A SWSAP1 ETS1 KRAS IL6R DHX40 RELA HIF1A TRIM32 FBXL17 LRRC59 ANTXR2 MYO10 ATXN1L CDC73 DYNC1I1 EN2 RAB5C CAB39 PSKH1 FBXO33 USP9X SCN1A PKIA SP3 C3orf18 BACH1 CHAF1A TM9SF3 RBMS3 RAPGEF2 ZNF236 CREBRF MORC3 PAXBP1 KBTBD2 MEIS1 WWC1 ZMYM2 MAP3K7 PALD1 RGP1 DUSP14 TAB2 TCF7L2 FAM168A HNRNPA3 CSNK1G2 ZNF518B CACUL1 MPEG1 LRP1B HBP1 NOVA1 XIAP FAM212B SOCS5 GPM6B VMA21 TAPT1 REPS2 MIDN ACTA1 SEMA5A TLE4 IL17RB STRN3 MAP3K10 ZSWIM6 TERF1 ITK LCA5 BSDC1 ZIC3 CSRNP2 CEBPB SGK3 TRPS1 BNC2 CSF1R OTUB2 RNF123 ARID2 TADA2B NDFIP1 GPD1L VAV3 RCN2 SATB1 BRD1 GABRA1 RPS6KA3 WEE1 CDX1 FXR1 ZNF652 RPS6KB1 PKN2 |
| miR-584 | 168 | KLHL13 PDHA1 CHORDC1 ZNF208 SRPK1 BMP3 UTRN USP6NL PPIL1 STARD13 PPM1A ARL13B FAXC HSF2 SRP72 DDHD2 PCDH18 GNG12 KIF2A ZNF583 COQ10B SH3PXD2A PRRX1 ZC3H6 TTC13 PITX1 SNX13 DAND5 ZNF627 NF1 IMPAD1 ZNF614 CD200 SLC30A7 NUDCD2 BTBD10 FAM122B ZBTB41 KIAA1841 PIGK ALPK1 WWP1 CEP104 SLC25A40 ANKRD18B RPE NAMPT C5orf51 THBS4 TNPO1 LRRTM2 OPCML FAM175B ANKRD44 CALCR ZNF585A RUNX2 MBNL3 RAP2A FLRT3 PURB ZNF512B SEMA4F HDAC1 FAM104B FNDC3B RNF11 GBP5 ITGAE ERRFI1 ADNP2 EEA1 XPO7 ACSL4 NBN TRAF3IP2 NUFIP2 MEX3B CALB1 AIDA ACYP2 BMPR1A PKD2 STAG2 MAML2 CTDSPL2 NPHP1 SFR1 PRPF19 MYO5B SPOPL HACE1 KRAS AQP4 GYPE PCDHB11 C18orf63 CALCA VPS41 PRKAA1 RLN1 THBD MORC3 TMTC3 AFF4 AKAP10 RAB23 PPP1R3B CGGBP1 SGTB MATN3 HBD SETD5 POLR3E USP45 CCNYL1 BCOR H2AFZ NANP RQCD1 ABHD17C XPR1 PKN2 M1AP UHRF1BP1 ZNF512 PHF6 SLC35D3 EXPH5 ATP6V1E2 FOXA1 WIPI1 MMAA AVPR1A ZNF674 ATPAF1 MSI2 UBE4B KMT2A DSG3 GABRA4 NAA30 TMX1 TMEM17 EIF2AK1 LMO3 HBS1L ZNF431 MYRIP SSX2IP PTP4A1 PIGW HSD11B1 ELL2 YKT6 ETNK1 SRSF5 CHN1 LARP4B MEX3C UTP11L LRP11 TMEM106B SAR1B TP63 HAPLN1 PUM1 CCDC88A |
